# PARP inhibitors chemopotentiate and synergize with cisplatin to inhibit bladder cancer cell survival and tumor growth

**DOI:** 10.1186/s12885-022-09376-9

**Published:** 2022-03-23

**Authors:** Sayani Bhattacharjee, Matthew J. Sullivan, Rebecca R. Wynn, Alex Demagall, Andrew S. Hendrix, Puneet Sindhwani, Firas G. Petros, Nagalakshmi Nadiminty

**Affiliations:** 1 Department of Urology, College of Medicine and Life Sciences, University of Toledo Health Science Campus, 3000 Arlington Avenue, Toledo, OH 43614 USA; 2 Department of Cancer Biology, College of Medicine and Life Sciences, University of Toledo Health Science Campus, 3000 Arlington Avenue, Toledo, OH USA; 3 Graduate Program in Cancer Biology, College of Medicine and Life Sciences, University of Toledo Health Science Campus, 3000 Arlington Avenue, Toledo, OH USA; 4 College of Medicine and Life Sciences, University of Toledo Health Science Campus, 3000 Arlington Avenue, Toledo, OH 43614 USA

**Keywords:** Bladder cancer, Urothelial carcinoma, PARP inhibition, Cisplatin, DNA damage repair, Combination therapy

## Abstract

**Background:**

Management of bladder cancer (BLCA) has not changed significantly in the past few decades, with platinum agent chemotherapy being used in most cases. Chemotherapy reduces tumor recurrence after resection, but debilitating toxicities render a large percentage of patients ineligible. Recently approved immunotherapy can improve outcomes in only a third of metastatic BLCA patients. Therefore, more options for therapy are needed. In this study, we explored the efficacy of PARP inhibitors (PARPi) as single agents or as combinations with platinum therapy.

**Methods:**

We treated BLCA cells with PARPi (olaparib, niraparib, rucaparib, veliparib, or talazoparib) alone or as the combination of cisplatin with PARPi. We then measured their survival, proliferation, apoptosis, as well as their ability to form colonies. BLCA xenografts in male SCID mice were treated similarly, followed by the assessment of their growth, proliferation, and apoptosis.

**Results:**

PARPi niraparib and talazoparib were effective in reducing BLCA cell survival as single agents. Combinations of Cisplatin with talazoparib and niraparib effectively reduced the survival of BLCA cells, while veliparib was not effective even at high concentrations. *In vivo*, the combinations of cisplatin with niraparib, rucaparib, or talazoparib reduced BLCA xenograft growth significantly.

**Conclusions:**

We provide evidence that PARPi can be effective against BLCA as single agents or as combinatorial therapy with cisplatin.

**Supplementary Information:**

The online version contains supplementary material available at 10.1186/s12885-022-09376-9.

## Background

Bladder cancer (BLCA) is the sixth most common cancer in the US and the ninth most common cancer worldwide [1, 2]. While incidence rates are lower in women compared with men, incidence is rising universally as tobacco use rates increase in developing countries [3]. Most newly diagnosed BLCAs are superficial; however, ~30% exhibit invasion past the bladder submucosa/mucosa, which constitutes muscle-invasive bladder cancer (MIBC) [4]. The initial treatment for non-muscle-invasive bladder cancer (NMIBC) involves surgical resection followed by adjuvant therapy. Nearly 75% of NMIBC cases recur while ~25% progress to more advanced disease [4]. Muscle invasion is known to be associated with a high risk of death from distant metastases. Even after radical cystectomy, MIBC patients develop metastases that often result in death [5]. BLCA is very expensive to treat due to the multiyear surveillance by cystoscopy that is required after tumor resection.

The previous decades have not seen significant changes in the management of BLCA, with the combination of methotrexate, vinblastine, doxorubicin, and cisplatin (MVAC) or gemcitabine with cisplatin being used as the systemic therapy of choice since 1985 [6–9]. Adjuvant or neo-adjuvant chemotherapy with platinum agents reduces recurrence following resection [10–12]; however, not many patients are eligible, and meaningful responses can only be achieved in half of eligible patients [13]. Chemotherapy also produces multiple severe grade 4 toxicities including ototoxicity, nephrotoxicity, hepatotoxicity, and neurotoxicity [12, 14], leading to dose reduction which compromises efficacy. New therapy options include the checkpoint inhibitors atezolizumab, pembrolizumab, durvalumab, avelumab, and nivolumab approved in 2016 and 2017 as breakthrough immunotherapy [15]. However, no more than 20-30% of patients with metastatic BLCA exhibit either a partial or complete response to immunotherapy and no reliable markers of response have been identified [16]. Hence, there is great need for more therapeutic options.

Genomic DNA that is damaged due to free radicals, mutagens, or carcinogens is repaired by the Poly (ADP-Ribose) Polymerase family proteins. Seventeen members of the PARP family are known, with PARP1 accounting for most DNA damage repair (DDR) events in malignant and non-malignant cells. PARP1 binds to single strand breaks (SSB) and recruits a cascade of DDR proteins. Recruitment of these proteins results in PARP dissociation from DNA and SSB repair [17]. In cells deficient in PARP, SSBs are transformed into double strand breaks (DSB), which engage other repair mechanisms, namely homologous recombination (HR) and non-homologous end rejoining (NHEJ) [18]. The BRCA genes are essential for HR to occur. Deficiency in BRCA as well as PARP leads to “synthetic lethality” in cells [19], which points to the attractive therapeutic opportunity to target cancers that lack BRCA genes selectively with PARP inhibitors (PARPi).

PARPi were thought to be of little value in cancers such as prostate and bladder owing to the fact that inheritable BRCA mutations occur with low frequency in such cancers. Nevertheless, recent reports indicate that the usefulness of PARPi can encompass other perturbations in the HR pathway [20–22]. Many HR pathway gene mutations may manifest as “BRCAness”, which can mimic loss of BRCA genes and synthetic lethality [23]. BLCA is characterized by somatic loss of function (LOS) mutations in ATM, CHEK1, CHEK2, RAD51, BRCA1, BRCA2, ATR, and FANCF genes [6]. TCGA analyses revealed that 34% of BLCA exhibit truncating or missense mutations in genes that confer sensitivity to PARPi [24–27]. Despite these promising signals, studies on the value of PARPi in BLCA are limited.

Few earlier studies offer definitive conclusions verifying the efficacy of PARPi in BLCA. Given that HR gene mutations amplify the effects of DNA damage caused by ionizing radiation or platinum drugs, PARPi may also exhibit efficacy as adjunctive therapy with cisplatin or carboplatin. The current study was aimed to compare 5 commercially available PARPi in BLCA cells and test their efficacy in combination with cisplatin. Understanding the mechanisms and the association of HR repair defects with sensitivity to PARPi can signal a breakthrough in BLCA therapy. Established BLCA cell lines were chosen for the study based on observations that BLCA cell lines replicate many genetic aberrations associated with BLCA tumor development [26]. Our results showed that PARPi suppress the survival and proliferation of BLCA cells as single agents and also synergize with cisplatin in reducing the survival of BLCA cells and xenografts, demonstrating that PARPi can be therapeutics of choice in BLCA.

## Methods

### Analysis of Mutations in HR genes

Publicly available databases containing mutational data for HR pathway genes were queried using COSMIC, The Cancer Genome Atlas, and cBioPortal (https://cancer.sanger.ac.uk/cosmic, https://cancergenome.nih.gov/, and http://www.cbioportal.org/). We sought to determine the correlation between mutations in DDR pathway genes and BLCA patient survival. The list of DDR genes for which we sought to determine mutational status is from [28] and the percentage of mutations found in those genes in BLCA tissues from the TCGA cohort are listed in Table 1. We also report the death rates from BLCA in the TCGA cohort based on mutation rates for each gene, where available.

**Table 1:** Results from data mining using GDC Data Portal, COSMIC, and cBioPortal are summarized here. The percentages of mutations in Direct DNA Repair genes and genes involved in Indirect DNA Stability as well as death rates in the TCGA BLCA tissue cohort are reported.

Direct DNA Repair	COSMIC	GDC Data Portal	cBioPortal	Death Rates (GDC Data Portal)
ATM	7.91%	15.63%	9%	Unavailable
ERCC2	7.46%	12.67%	10%	Unavailable
BRCA2	6.09%	12.40%	7%	Unavailable
ATR	1.19%	10.24%	5%	Unavailable
PRKDC	0	9.43%	8%	Unavailable
POLE	1.49%	7.28%	5%	Unavailable
FANCD2	1.64%	7.01%	9%	Unavailable
FANCA	2.24%	6.74%	6%	Unavailable
SLX4	1.64%	6.47%	5%	Unavailable
FANCM	1.49%	6.20%	4%	Unavailable
BRIP1	1.64%	5.93%	5%	Unavailable
BRCA1	1.94%	5.66%	5%	Unavailable
OGG1	0.30%	3.50%	7%	Unavailable
NBN	1.19%	3.23%	6%	Unavailable
RAD54B	1.04%	1.89%	6%	Unavailable
DCLRE1C	0.30%	1.89%	5%	Unavailable
XPC	0.30%	1.62%	5%	Unavailable
NEIL2	0.15%	1.35%	6%	Unavailable
**Indirect DNA Stability**				
TP53	28%	55.26%	48%	44.39%
POLQ	1.49%	10.24%	5%	36.84%
CDK12	1.64%	8.63%	6%	37.50%
REV3L	1.49%	7.55%	5%	32.14%
TP53BP1	1.34%	7.01%	5%	26.92%
CENPE	2.39%	6.47%	4%	41.67%
MDC1	2.09%	6.47%	5%	33.33%
KNTC1	1.34%	5.39%	5%	40%
BUB1	1.04%	5.12%	5%	21.05%
RNF168	1.34%	4.04%	5%	26.67%
BAP1	1.34%	4.04%	5%	40%
RECQL4	0.45%	4.04%	5%	40%
POLN	1.04%	3.77%	5%	28.57%
CLK2	0.30%	3.50%	5%	23.08%
WRN	1.19%	2.96%	5%	36.36%
RRM2B	0	1.35%	9%	40%
TDP2	0.15%	1.08%	5%	25%
RAD18	0.45%	0.81%	6%	33.33%
RAD1	0.30%	0.81%	6%	100%
POLB	0	0.27%	5%	0%

### Cell lines and other reagents

UM-UC-3, T-24 (human bladder cancer cell lines), and SV-HUC-1 (normal human bladder epithelial cell line) were obtained from the American Type Culture Collection (ATCC, Manassas, VA) and were cultured in EMEM, McCoy’s 5a, or F12K media respectively, supplemented with 10% FBS and penicillin/streptomycin. Cells were used within half a year after being received from ATCC or after thawing from cryopreservation. Short Tandem Repeat (STR) profiling is used by the ATCC for cell line authentication. The cell lines in culture were routinely tested for mycoplasma contamination every two months using the MycoFluor^TM^ Mycoplasma detection kit (Thermo Fisher Scientific, Waltham, MA). Tubulin antibodies were obtained from Thermo Fisher Scientific, Waltham, MA. Cleaved and whole caspases 3, 7, and 9 antibodies were obtained from Cell Signaling Technology, Danvers, MA. Ki-67 antibodies were obtained from Neomarker, Fremont, CA. The PARP inhibitors, olaparib, niraparib, rucaparib, veliparib, and talazoparib, were obtained from MedChem Express, Monmouth Junction, NJ. Cisplatin was from Sigma Aldrich, St. Louis, MO. Other reagents were supplied by local suppliers such as Fisher Scientific and VWR International.

### Assays for Cell Viability

SV-HUC-1, UM-UC-3, or T-24 cells were seeded at 1000 cells/well in 96-well plates and treated with PARPi or their combinations with cisplatin as shown in the respective figures. A Coulter cell counter (Beckman Coulter, Indianapolis, IN) was used to determine cell viability.

### Assays for Cell Proliferation

SV-HUC-1, UM-UC-3, or T-24 cells were seeded at 1000 cells/well in 96-well plates and treated with PARPi or their combinations with cisplatin for 3 days. The CellTiter 96® Aqueous One Solution Cell Proliferation kit (Promega, Madison, WI) was used according to manufacturer instructions to assess cell proliferation.

### Protein Analysis by Western Blotting

High salt buffer containing 50 mM Hepes pH 7.9, 250 mM NaCl, 1 mM EDTA, 1% NP-40, 1 mM PMSF, 1 mM Na Vanadate, 1 mM NaF, and protease inhibitors (Roche) was used to lyse cells as described earlier [29]. The Coomassie Protein Assay Reagent (Pierce) was used to measure total amounts of protein. Total proteins (30-40 μg) were resolved on 10% SDS–PAGE followed by transfer to nitrocellulose membranes. Subsequently, the blots were blocked in 5% nonfat milk diluted in PBST (1x PBS+0.1% Tween-20) for 1 h and incubated overnight with primary antibodies diluted in 1% BSA. ECL (Millipore) was used for signal detection after the blots were incubated with the respective HRP-conjugated secondary antibodies. Image J was used to calculate band intensities.

### Assays for Clonogenicity

Anchorage-dependent clonogenicity was assayed as described in earlier studies [30]. SV-HUC-1, UM-UC-3, or T-24 cells were seeded at 30,000 cells/well in 12-well plates and treated with different PARPi concentrations or their combinations with cisplatin for 3 days. Cells were trypsinized and replated at low density (400 cells in each well) in triplicate in 6-well multiwell plates. Cells were left undisturbed with no media changes at 37^o^C for 10-14 days. The colonies were stained with 0.5% Crystal Violet in buffered formalin and colony numbers were counted using ImageJ.

### In vivo xenografts in mice

Mouse xenograft models are commonly used to assess the efficacy of therapeutic strategies. A total of 60 male 4–5-week-old SCID mice (Charles River, Wilmington, MA) with an average weight of 20 g were used in this study. Mice were allowed to acclimate for 7 days after receipt from the vendor and were housed at 22.5 ± 0.5^o^C in sterile cages. We injected 2 million UM-UC-3 cells sub-cutaneously in a 1:1 (v/v) ratio with matrigel into both flanks of mice and monitored tumor growth. After average tumor volumes reached 0.1 cm^3^ approximately, we divided the mice randomly into 10 groups (*n *= 5/group). The treatment groups were: 1) 0.5% Methocel A4M as vehicle control, 2) niraprib at 10 mg/kg, 3) olaparib at 25 mg/kg, 4) rucaparib at 50 mg/kg, 5) talazoparib at 0.5 mg/kg delivered via daily oral gavage, 6) cisplatin at 0.5 mg/kg delivered intra-peritoneally once every two days, 7) cisplatin+niraparib, 8) cisplatin+olaparib, 9) cisplatin+rucaparib, or 10) cisplatin+talazoparib. Mice were treated for three weeks and growth of the tumors and mouse weights were measured using digital calipers or a balance every other day. Tumor growth was used as the outcome measure. When the control tumors reached an average of 1500 mm^3^, mice in all groups were euthanized with carbon dioxide followed by cervical dislocation. No animals were excluded from any analyses. Tumor inhibition was calculated as percentage tumor growth inhibition compared with vehicle control. We harvested the xenograft tissues and analyzed the expression of ki-67, cleaved caspases 3, 7, and 9 with immunohistochemistry. All experiments with animals were governed by the Institutional Animal Care and Use Committee of the University of Toledo (IACUC protocol # 108804) and were performed in line with the National Institutes of Health Guide for the Care and Use of Laboratory Animals.

### Immunohistochemistry

Immunohistochemistry was performed as described earlier [31]. Tumor tissues were fixed in formalin and paraffin-embedded tissue blocks were cut into 5-micron sections. Sections were dewaxed and rehydrated followed by blocking of endogenous peroxidase activity. Sodium citrate buffer (0.01 mol/L, pH 6.0) was used for antigen retrieval in a microwave at 1,000 W for 3 min followed by 100 W for 20 min. Nonspecific antibody binding was blocked by incubation in 10% fetal bovine serum in PBS for 30 min at room temperature. This was followed by incubation with 1:500 dilution of Ki-67 (NeoMarkers, Fremont, CA), Cleaved Caspase-3, Cleaved Caspase-7, or Cleaved Capase-9 (Cell Signaling Technology, Danvers, MA) antibodies overnight at 4 °C (Suppl. Fig. 1). The sections were subsequently incubated with biotin-conjugated secondary antibodies for 30 min, and with avidin DH-biotinylated horseradish peroxidase complex for 30 min (Vectastain ABC Elite Kit, Vector Laboratories). Signal development was achieved using the diaminobenzidine substrate kit (Vector Laboratories, Burlingame, CA). Sections were then counterstained with hematoxylin and coverslipped. Signal intensity was quantified semi-quantitatively using the ImageJ Fiji software as detailed earlier [32, 33]. Briefly, the Color Deconvolution plug-in of the ImageJ Fiji software was used to digitally separate the DAB and hematoxylin signals. The DAB signal was measured as mean gray values with the upper and lower thresholds set at 200 and 120. Then the Analyze Particle Numbers function was used to determine the number of nuclei in the same field from the hematoxylin image. The mean gray values of DAB staining were then normalized by the number of nuclei in each field. The signal intensity for ki-67, cleaved caspase-3, cleaved caspase-7, or cleaved caspase-9 was determined as the average of signal intensities measured from 5 different images per marker. The data are presented in dot plots with the signal intensities measured in each of the images shown along with the means.

### Analyses for statistical significance

Results are reported as means ± SD. One-way ANOVA was used for the comparison of multiple groups with alpha set at 0.05. A P value cut-off ≤0.05 was established to indicate significance. All data were analyzed using the Microsoft Excel Data Analysis Toolpak for Windows 10 (Microsoft, Seattle, WA).

## Results

### BLCA patient tumors from the TCGA cohort have mutations in DDR genes

We used GDC Data Portal, COSMIC, and cBioPortal to analyze mutation rates in DNA repair genes [28] in BLCA patient tumors from the TCGA. The data revealed that ATM, ERCC2, BRCA2, ATR, and TP53 mutations are highly prevalent in BLCA tissues from the TCGA cohort (Table 1). Mutations in genes involved in indirect DNA stability were also associated with high death rates from BLCA in this cohort (Table 1). These data confirmed previous findings which showed that ~34% of BLCA harbor mutations in DDR genes [24, 34, 35]. The results provided the rationale for our study to test the relative efficacy of commercially available PARPi against BLCA cells.

### PARPi suppress cell survival of BLCA cells

**Fig. 1:**
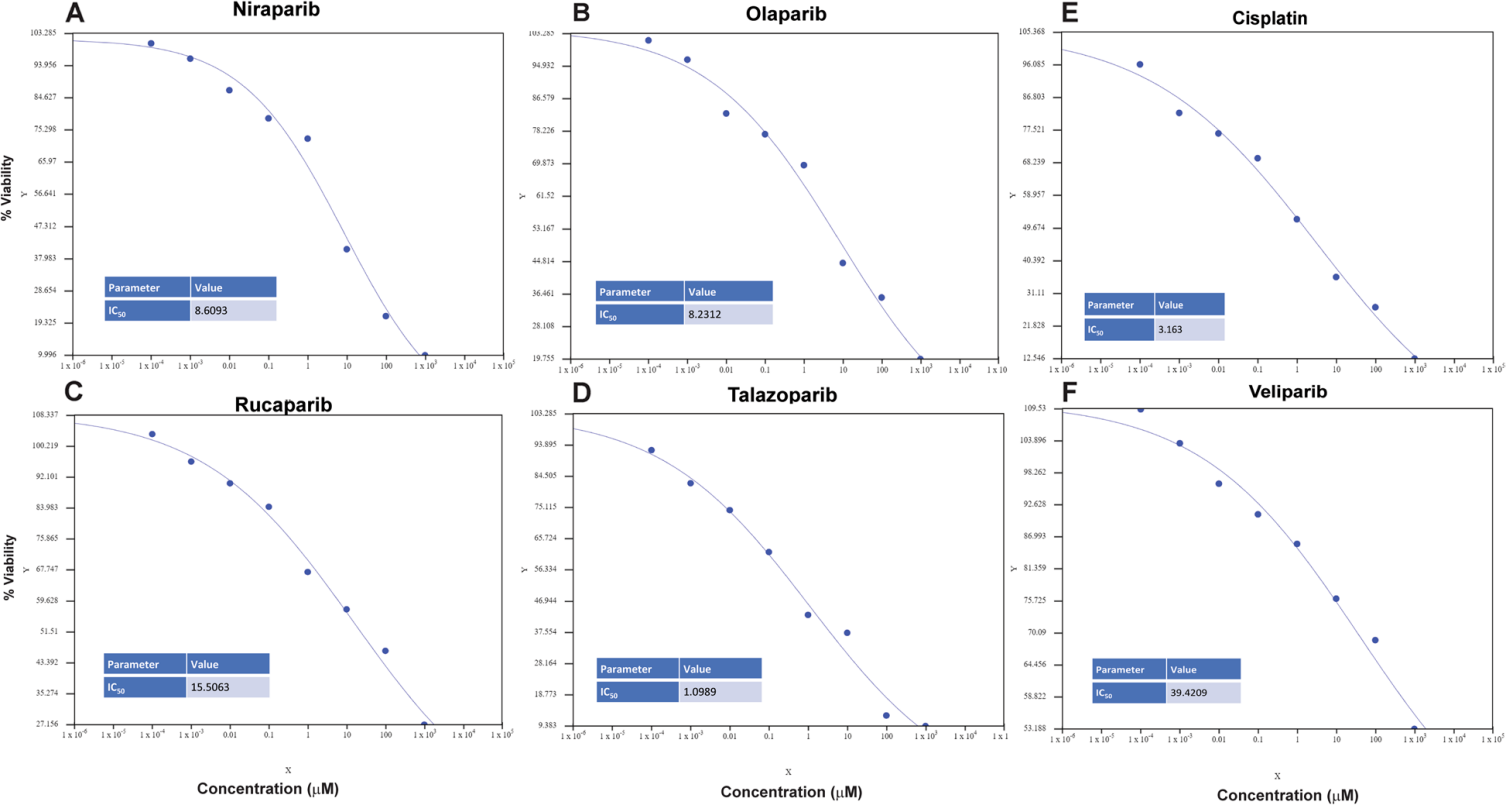
UM-UC-3 cells were treated with 0, 0.0001, 0.001, 0.01, 0.1, 1, 10, 100, or 1000 μM concentrations of niraparib, olaparib, rucaparib, talazoparib, veliparib, or cisplatin for 72 h. Cell survival was reported as % cells surviving compared with vehicle-treated control. IC_50_s were calculated using the AAT Bioquest Online IC_50_ calculator

We determined the IC_50_ of cisplatin as well as the PARPi (olaparib, niraparib, rucaparib, veliparib, and talazoparib) in UM-UC-3 cells by treating with 0, 0.0001, 0.001, 0.01, 0.1, 1, 10, 100, and 1000 μM concentrations for 72 h. The AAT Bioquest (https://www.aatbio.com/tools/ic50-calculator) online tool was used to calculate IC_50_ values. The IC_50_ curves and the calculated IC_50_ values for each of these agents are summarized in Fig. 1A-F. IC_50_ values (μM) were as follows: Niraparib (8.6093); olaparib (8.2312); rucaparib (15.5063); talazoparib (1.0989); veliparib (39.4209); and cisplatin (3.163).

**Fig. 2:**
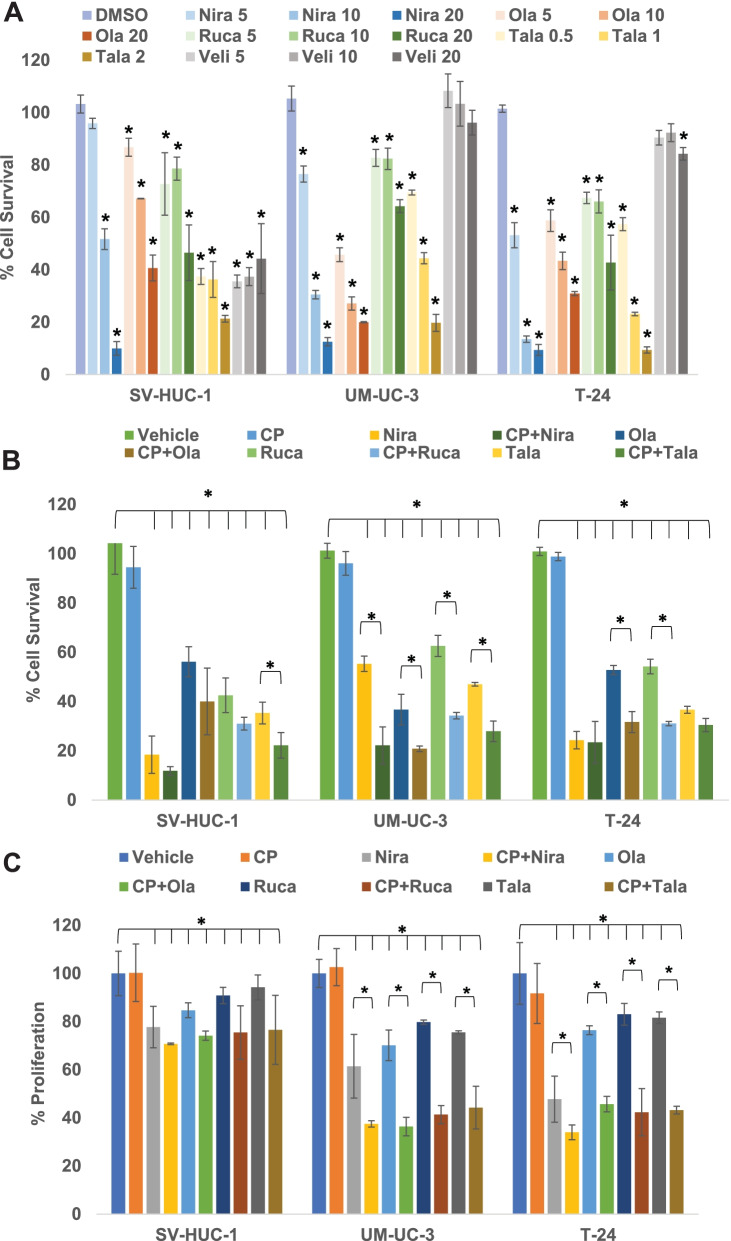
PARPi suppress the cell survival and proliferation of BLCA cells *in vitro*. **A** The BLCA cell lines UM-UC-3 and T-24 and the normal urothelial cells SV-HUC-1 were treated with varying concentrations of PARPi (niraparib, olaparib, rucaparib, talazoparib, or veliparib) for 72 h to determine the effective concentrations to be used in subsequent assays. Cell survival was measured as the percentage of cells surviving in comparison with the DMSO vehicle control in each cell line. **B** The BLCA cell lines UM-UC-3 and T-24 and the normal urothelial cells SV-HUC-1 were treated with sub-IC_50_ concentrations of niraparib, olaparib, rucaparib (5 μM each), talazoparib (0.5 μM) either singly or in combination with sub-IC_50_ concentration of cisplatin (0.5 μM) for 72 h. Cell survival was measured as the percentage of cells surviving in comparison with DMSO vehicle control in each cell line. **C** The BLCA cell lines UM-UC-3 and T-24 and the normal urothelial cells SV-HUC-1 were treated with sub-IC_50_ concentrations of niraparib, olaparib, rucaparib (5 μM each), talazoparib (0.5 μM) either singly or in combination with sub-IC_50_ concentration of cisplatin (0.5 μM) for 72 h. Cell proliferation was measured as the percentage of cells proliferating in comparison with DMSO vehicle control in each cell line. All results are presented as means±SD of 3 independent experiments with triplicates. P ≤ 0.05 was considered significant (*)

Next, to determine working concentrations for each of the PARPi, we treated UM-UC-3 and T-24 as well as SV-HUC-1 cells with 5, 10, or 20 μM of olaparib, niraparib, veliparib, or rucaparib or 0.5, 1, or 2 μM of talazoparib for 3 days. As demonstrated in Fig. 2A, olaparib, niraparib, talazoparib, and rucaparib significantly blocked the survival of UM-UC-3, T-24, and SV-HUC-1 cells. Veliparib did not achieve >30% inhibition of BLCA cell survival even at very high concentrations. Our findings indicate that talazoparib and niraparib achieved >50% reduction in survival of BLCA cells at low concentrations.

### PARPi reduce proliferation of BLCA cells

We treated BLCA cell lines UM-UC-3 and T-24 and the normal urothelial cells SV-HUC-1 with sub-IC_50_ concentrations of PARPi for 5 days. As shown in Fig. 2C, the percentages of proliferation in niraparib-treated SV-HUC-1, UM-UC-3, and T-24 cells were 77.75 ± 8.63 (*p* = 0.034), 61.43 ± 13.24 (*p* = 0.011), and 47.71 ± 9.58 (*p* = 0.0012), respectively, compared with their respective DMSO-treated controls. The percentages of proliferation in olaparib-treated SV-HUC-1, UM-UC-3, and T-24 cells were 84.67 ± 3.10 (*p* = 0.041), 70.11 ± 6.36 (*p* = 0.035), and 76.39 ± 1.84 (*p* = 0.022), respectively, compared with their respective DMSO-treated controls. The percentages of proliferation in rucaparib-treated SV-HUC-1, UM-UC-3, and T-24 cells were 90.84 ± 3.41 (*p* = 0.07), 79.78 ± 0.85 (*p* = 0.039), and 83.03 ± 4.51 (*p* = 0.046), respectively. The percentages of proliferation in talazoparib-treated SV-HUC-1, UM-UC-3, and T-24 cells were 94.22 ± 5.18 (*p* = 0.43), 75.54 ± 0.66 (*p* = 0.036), and 81.61 ± 2.42 (*p* = 0.047), respectively, compared with their respective DMSO-treated controls. These results indicated that PARPi suppressed the proliferation of UM-UC-3 and T-24 more significantly compared with that of SV-HUC-1 cells, suggesting that PARPi can be used as potential therapeutic agents against BLCA.

### PARPi inhibit the clonogenic ability of BLCA cells

**Fig. 3:**
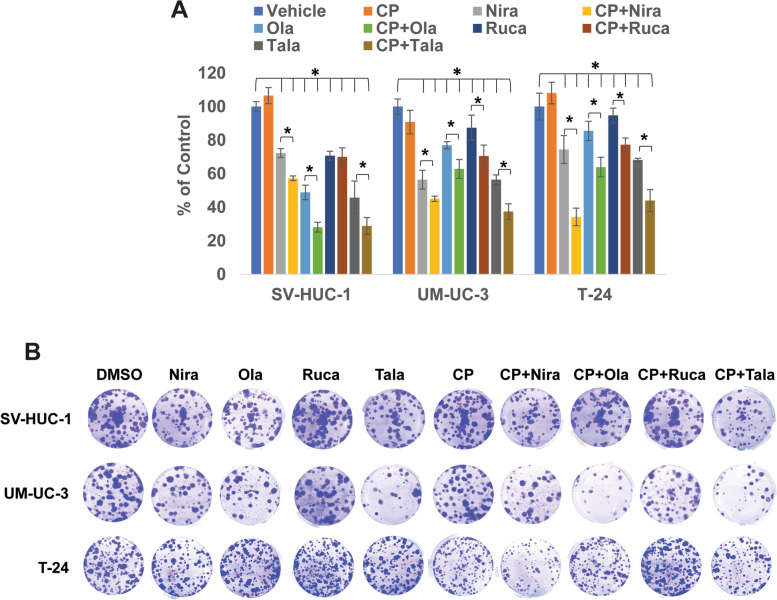
PARPi suppress the clonogenic ability of BLCA cells *in vitro*. **A** The BLCA cell lines UM-UC-3 and T-24 and the normal urothelial cells SV-HUC-1 were treated with sub-IC_50_ concentrations of niraparib, olaparib, rucaparib (5 μM each), talazoparib (0.5 μM) either singly or in combination with sub-IC_50_ concentration of cisplatin (0.5 μM) for 72 h. Cells were plated at low densities (400 cells/well) in 6-well plates and incubated at 37^o^C in a 5% CO_2_-incubator for 10-14 days. At the end of the experiment, colonies were stained with 0.5% crystal violet in buffered formalin and counted using the Colony Counter plug-in of ImageJ. Results are presented as means±SD of 3 independent experiments with triplicates. *P* ≤ 0.05 was considered significant (*). **B** Representative images of colonies formed by SV-HUC-1, UM-UC-3, and T-24 cells after treatment

We treated BLCA cell lines UM-UC-3 and T-24 as well as the normal urothelial cells SV-HUC-1 with sub-IC_50_ concentrations of PARPi for 72 h and performed clonogenic assays as described earlier [31]. The percentages of colonies formed in niraparib-treated SV-HUC-1, UM-UC-3, and T-24 groups were 72.30 ± 2.66 (*p* = 0.036), 56.44 ± 5.62 (*p* = 0.022), and 74.43 ± 8.30 (*p* = 0.013), respectively, compared with their DMSO-treated controls. The percentages of colonies formed in olaparib-treated SV-HUC-1, UM-UC-3, and T-24 groups were 48.84 ± 4.36 (*p* = 0.0021), 76.99 ± 2.12 (*p* = 0.037), and 85.52 ± 5.75 (*p* = 0.047), respectively, compared with their DMSO-treated controls. The percentages of colonies formed in rucaparib-treated SV-HUC-1, UM-UC-3, and T-24 groups were 70.76 ± 2.66 (*p* = 0.033), 87.42 ± 7.53 (*p* = 0.048), and 94.73 ± 4.4 (*p* = 0.71), respectively, compared with their respective DMSO-treated controls. The percentages of colonies formed in talazoparib-treated SV-HUC-1, UM-UC-3, and T-24 groups were 45.76 ± 9.81 (*p* = 0.0019), 56.44 ± 2.81 (*p* = 0.0031), and 68.23 ± 0.97 (*p* = 0.002), respectively, compared with their respective DMSO-treated controls. The results showed that PARPi inhibited the clonogenic ability of BLCA cells and normal urothelial cells significantly (Fig. 3A, B), indicating that PARPi may suppress the ability of BLCA cells to recover from treatment and form colonies.

### PARPi synergize with cisplatin treatment in vitro

Cisplatin is the mainstay of BLCA therapy. However, cisplatin treatment produces life-threatening toxicities in ~50% of BLCA patients. Strategies to overcome these drawbacks are needed urgently. Towards this end, we sought to determine whether co-treatment with PARPi can be used to reduce the effective dosage of cisplatin against BLCA cells. Hence, we treated BLCA cells UM-UC-3 and T-24 as well as the normal urothelial cells SV-HUC-1 with sub-IC_50_ concentrations of PARPi in combination with sub-IC_50_ concentrations of cisplatin and measured cell survival, proliferation, and clonogenic ability of the treated cells compared with vehicle-treated cells. The percentages of cells surviving in cisplatin-treated SV-HUC-1, UM-UC-3, and T-24 groups were 94.46 ± 8.5 (*p* = 0.38), 96.09 ± 4.8 (*p* = 0.42), and 98.85 ± 1.66 (*p* = 0.31), respectively, compared with their DMSO-treated controls. The percentages of cells surviving in niraparib-treated vs. CP+Niraparib-treated SV-HUC-1, UM-UC-3, and T-24 groups were 18.44 ± 7.56 vs. 11.85 ± 1.74 (*p* = 2.663e-8), 55.34 ± 3.10 vs. 22.19 ± 7.56 (*p* = 1.57e-6), and 24.33 ± 3.53 vs. 23.44 ± 8.49 (*p* = 0.00059), respectively, compared with their respective DMSO-treated controls. The percentages of cells surviving in olaparib-treated vs. CP+Olaparib-treated SV-HUC-1, UM-UC-3, and T-24 groups were 56.14 ± 6.08 vs. 40.03 ± 3.54 (*p* = 3.69e-4), 36.75 ± 6.22 vs. 20.84 ± 1.08 (*p* = 1.22e-5), and 52.83 ± 1.86 vs. 31.69 ± 4.21 (*p* = 3.19e-3), respectively, compared with their respective DMSO-treated controls. The percentages of cells surviving in rucaparib-treated vs. CP+Rucaparib-treated SV-HUC-1, UM-UC-3, and T-24 groups were 42.53 ± 7.03 vs. 31.04 ± 2.57 (*p* = 4.17e-4), 62.60 ± 4.29 vs. 34.29 ± 1.32 (*p* = 3.81e-4), and 54.20 ± 2.93 vs. 31.05 ± 0.86 (*p* = 6.43e-4), respectively, compared with their respective DMSO-treated controls. The percentages of cells surviving in talazoparib-treated vs. CP+Talazoparib-treated SV-HUC-1, UM-UC-3, and T-24 groups were 35.35 ± 4.38 vs. 22.22 ± 5.20 (*p* = 4.21e-6), 47.01 ± 0.69 vs. 27.95 ± 4.16 (*p* = 0.0014), and 36.63 ± 1.41 vs. 30.48 ± 2.67 (*p* = 2.19e-3), respectively, compared with their respective DMSO-treated controls. The percentages of cell proliferation in niraparib-treated vs. CP+Niraparib-treated SV-HUC-1, UM-UC-3, and T-24 groups were 77.75 ± 8.63 vs. 70.74 ± 0.32 (*p* = 0.049), 61.43 ± 13. 24 vs. 37.49 ± 1.30 (*p* = 0.031), and 47.71 ± 9.58 vs. 33.97 ± 3.06 (*p* = 0.015), respectively, compared with their respective DMSO-treated controls. The percentages of cell proliferation in olaparib-treated vs. CP+Olaparib-treated SV-HUC-1, UM-UC-3, and T-24 groups were 84.67 ± 3.10 vs. 74.14 ± 1.89 (*p* = 0.06), 70.11 ± 6.36 vs. 36.39 ± 3.83 (*p* = 0.037), and 76.39 ± 1.84 vs. 45.69 ± 3.24 (*p* = 0.022), respectively, compared with their respective DMSO-treated controls. The percentages of cell proliferation in rucaparib-treated vs. CP+Rucaparib-treated SV-HUC-1, UM-UC-3, and T-24 groups were 90.84 ± 3.41 vs. 75.45 ± 11.12 (*p* = 0.072), 79.78 ± 0.85 vs. 41.35 ± 3.77 (*p* = 0.043), and 83.03 ± 4.51 vs. 42.32 ± 9.77 (*p* = 0.044), respectively, compared with their respective DMSO-treated controls. The percentages of cell proliferation in talazoparib-treated vs. CP+Talazoparib-treated SV-HUC-1, UM-UC-3, and T-24 groups were 94.22 ± 5.18 vs. 76.56 ± 14.34 (*p* = 0.069), 75.54 ± 0.66 vs. 44.24 ± 8.88 (*p* = 0.016), and 81.61 ± 2.42 vs. 43.22 ± 1.6 (*p* = 0.0449), respectively, compared with their respective DMSO-treated controls. The percentages of colonies formed in niraparib-treated vs. CP+Niraparib-treated SV-HUC-1, UM-UC-3, and T-24 groups were 72.30 ± 2.66 vs. 57.30 ± 1.33 (*p* = 0.044), 56.44 ± 5.62 vs. 45.09 ± 1.59 (*p* = 0.021), and 74.43 ± 8.3 vs. 34.21 ± 5.23 (*p* = 0.031), respectively, compared with their respective DMSO-treated controls. The percentages of colonies formed in olaparib-treated vs. CP+Olaparib-treated SV-HUC-1, UM-UC3, and T-24 groups were 48.84 ± 4.36 vs. 28.07 ± 2.90 (*p* = 0.002), 76.99 ± 2.12 vs. 62.88 ± 5.62 (*p* = 0.023), and 85.52 ± 5.75 vs. 63.90 ± 5.86 (*p* = 0.049), respectively, compared with their respective DMSO-treated controls. The percentages of colonies formed in rucaparib-treated vs. CP+Rucaparib-treated SV-HUC-1, UM-UC-3, and T-24 groups were 70.76 ± 2.66 vs. 70 ± 5.45 (*p* = 0.073), 87.42 ± 7.53 vs. 70.55 ± 6.46 (*p* = 0.066), and 94.73 ± 4.40 vs. 77.25 ± 3.94 (*p* = 0.048), respectively, compared with their respective DMSO-treated controls. The percentages of colonies formed in talazoparib-treated vs. CP+Talazoparib-treated SV-HUC-1, UM-UC-3, and T-24 groups were 45.76 ± 9.81 vs. 28.84 ± 5.02 (*p* = 2.47e-5), 56.44 ± 2.81 vs. 37.42 ± 4.61 (*p* = 1.22e-3), and 68.23 ± 0.97 vs. 43.98 ± 6.50 (*p* = 0.0031), respectively, compared with their respective DMSO-treated controls. The combinations of PARPi with sub-IC_50_ concentrations of cisplatin inhibited cell survival (Fig. 2B), proliferation (Fig. 2C), and clonogenic ability (Fig. 3A, B) of BLCA cells significantly, compared with the effects observed as single agents. The combination treatments also inhibited the survival, proliferation, and clonogenicity of normal urothelial cells. These results implied that PARPi may synergize with cisplatin in inhibiting the growth and survival of BLCA cells and effectively reduce the amount of cisplatin needed for anti-cancer effects.

### PARPi induce apoptosis in BLCA cells in vitro

**Fig. 4:**
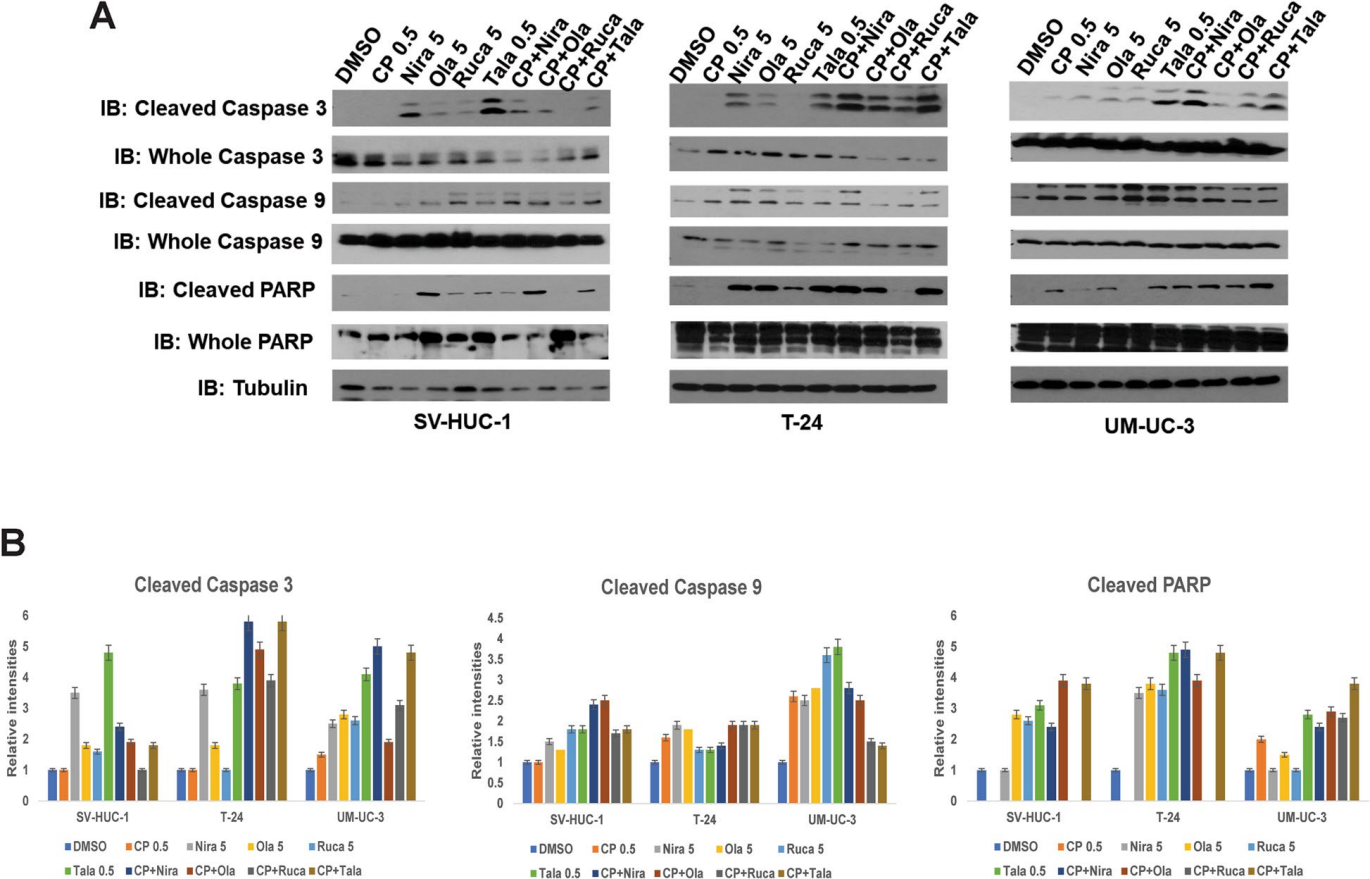
PARPi induce apoptosis in BLCA cells *in vitro*. **A** The BLCA cell lines UM-UC-3 and T-24 and the normal urothelial cells SV-HUC-1 were treated with sub-IC_50_ concentrations of niraparib, olaparib, rucaparib (5 μM each), talazoparib (0.5 μM) either singly or in combination with sub-IC_50_ concentration of cisplatin (0.5 μM) for 72 h. The resulting cell lysates were subjected to Western blotting with antibodies against the apoptotic markers cleaved/whole caspase 3, cleaved/whole caspase 9, or cleaved/whole PARP. Representative images of 3 independent experiments with duplicates are presented. **B** Relative band intensities (compared to whole caspase/PARP and Tubulin) were measured using ImageJ and are presented for Cleaved caspases 3 and 9 and cleaved PARP

We treated BLCA cell lines UM-UC-3 and T-24 as well as the normal urothelial cells SV-HUC-1 with sub-IC_50_ concentrations of PARPi either alone or in combination with sub-IC_50_ concentration of cisplatin for 72 h and subjected the resulting cell lysates to Western blotting with the apoptosis markers cleaved caspases 3 and 9, or cleaved PARP. As shown in Fig. 4, cells treated with the combination of cisplatin and PARPi showed higher levels of caspase cleavage as well as that of PARP, indicating that PARPi not only suppress growth and proliferation of BLCA cells but also induce apoptosis.

### In vivo synergism between PARPi and cisplatin

**Fig. 5:**
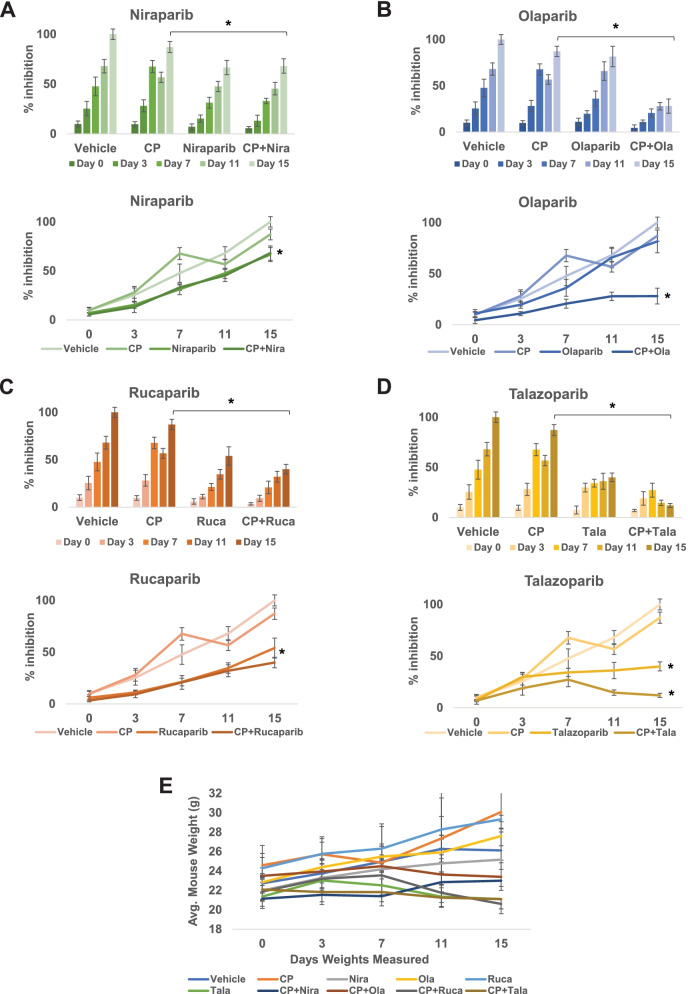
PARPi suppress the growth of BLCA xenografts *in vivo*. Xenografts of UM-UC-3 cells in SCID mice were generated by injecting 2x10^6^ cells into both flanks of SCID mice (*n* = 5/group). When the tumor volumes reached ~100 mm^3^, mice were treated with the PARPi niraparib (**A**), olaparib (**B**), rucaparib (**C**), talazoparib (**D**) or a combination of PARPi with cisplatin as detailed in the Methods section. Tumor growth was monitored using digital calipers. Toxicity was assessed by weighing the mice twice a week. At the end of the experiment, tumor tissues were harvested, and tumor inhibition was calculated as percentage tumor growth inhibition compared with vehicle control. **E** Average body weights±SD of all mice in each group are shown

**Fig. 6:**
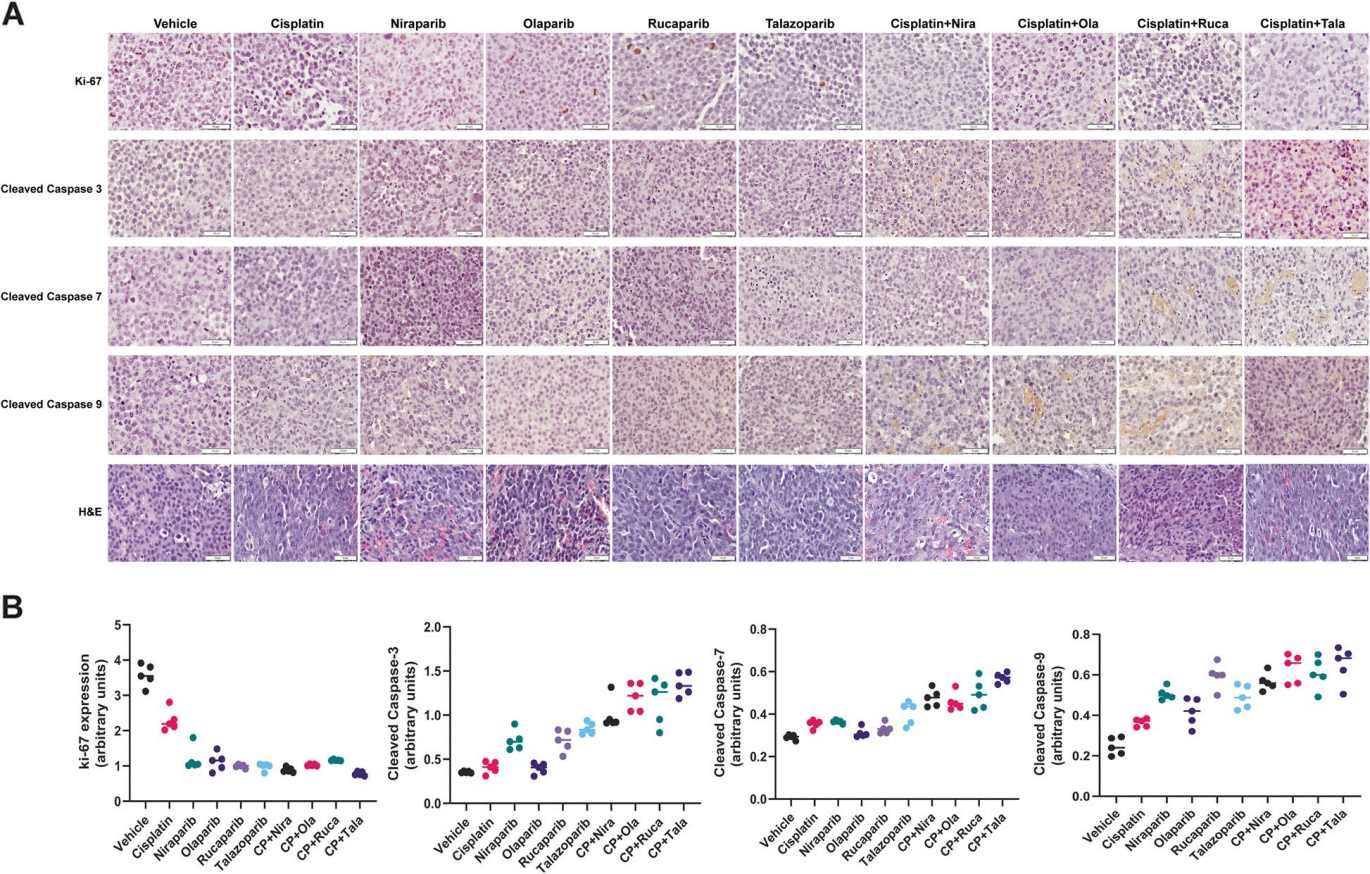
PARPi induce apoptosis and suppress proliferation in BLCA xenografts *in vivo*. Xenografts of UM-UC-3 cells in SCID mice were generated by injecting 2x10^6^ cells into both flanks of SCID mice. When the tumor volumes reached ~100 mm^3^, mice were treated with the PARPi niraparib, olaparib, rucaparib, talazoparib, or a combination of PARPi with cisplatin as detailed in the Methods section. At the end of the experiment, tumor tissues were harvested, and sections prepared for immunohistochemistry with antibodies against the proliferation marker ki-67, and the apoptotic markers cleaved caspases 3, 7, or 9. **A** Representative images from 10 tumors in each group are presented. The scale bar at the lower right corner in each panel represents 50 μm. **B** Staining intensities were calculated as detailed in Methods using the ImageJ Fiji software. Dot plots showing staining intensities in arbitrary units are presented

To establish that PARPi can synergize with cisplatin treatment *in vivo*, we generated xenografts of UM-UC-3 cells in male SCID mice followed by treatment with PARPi (olaparib, niraparib, talazoparib, or rucaparib), cisplatin, or cisplatin+different PARPi [6]. Toxicity was monitored by examining weight loss and serum ALP or AST activity. The xenografts were treated for 3 weeks and tumor tissues were collected. Immunohistochemistry was used to examine FFPE tumor sections for ki-67 and caspases 3, 7, and 9 to measure proliferation and apoptosis in the tumor tissues, respectively. At the end of the experiment, the vehicle control tumors measured 2287.021 ± 150.51 mm^3^. Niraparib (alone 1086.25 ± 76.2 mm^3^, *p* = 0.013 vs. CP+Niraparib 1037.48 ± 77.9 mm^3^, *p* = 0.034) and rucaparib (alone 1233.75 ± 61.65 mm^3^, *p* = 0.046 vs. CP+Rucaparib 1023.54 ± 64.21 mm^3^, *p* = 0.037) reduced tumor growth to a similar extent singly as well as in combination with cisplatin (Fig. 5A and C). On the other hand, olaparib showed strongly additive effects in reducing tumor growth when used in combination with cisplatin, while not being very effective as a single agent (olaparib alone 1865.63 ± 93.25 mm^3^, *p* = 0.07 vs. CP+Olaparib 641.28 ± 32.05 mm^3^, *p* = 0.002) (Fig. 5B). Talazoparib not only reduced tumor growth as a single agent but inhibited tumor growth virtually completely when used in combination with cisplatin (talazoparib alone 824.57 ± 41.2 mm^3^, *p* = 0.0061 vs. CP+Talazoparib 333.95 ± 16.65 mm^3^, *p* = 0.0018) (Fig. 5D). The P values shown are in comparison with the vehicle-treated tumors. There were no significant differences in average mouse weights between different treatment groups (Fig. 5E and Suppl. Fig. 1). ALP and AST activities in the sera, denoting potential hepatic injury, were not significantly different between the different treatment groups (Suppl. Fig. 2). Immunohistochemistry analyses demonstrated that tumor proliferation was suppressed in the PARPi and cisplatin combination treatments, while induction of apoptosis is evidenced by higher levels of cleaved caspases in the xenografts treated with the PARPi and cisplatin combinations (Fig. 6A and B).

## Discussion

Cisplatin-based chemotherapy has been used as the first line therapy for locally advanced BLCA for decades [36]. However, nearly 50% of patients progress on cisplatin-based therapy and approximately a third of patients are ineligible due to comorbidities [37]. Hence, there is a great need for the identification of new combinatorial strategies.

PARP inhibition relies on the presence of mutations or alterations in DNA damage genes such as those involved in HR. In-silico analyses revealed that ~30% of BLCA harbor mutations in HR genes, indicating that PARP inhibition can be used in BLCA. Earlier studies showed that PARP inhibition combined with cisplatin significantly increased lifespan and restored nerve conduction velocity in animal models [38]. PARP inhibition in combination with carboplatin and paclitaxel significantly improved progression-free survival in ovarian cancer patients [39]. PARP inhibitors can also protect against dose-limiting toxicity seen with some cancer therapies [40]. Several clinical trials are exploring PARP inhibitor combinations with platinum drugs in breast, lung, or ovarian cancers (NCT02595905; NCT01074970; NCT01086254; NCT04728230; NCT01345357; NCT02855944). Clinical trials examining the efficacy of PARP inhibitors in BLCA have started recruiting patients (NCT03375307). A few clinical trials such as NCT02546661, NCT03534492, and NCT03459846 are exploring the combination of PARP inhibitors with immunotherapy. In this study, we aimed to compare the relative efficacy of the 5 commercially available PARP inhibitors against BLCA cells and the value of combining them with cisplatin. In vitro studies to test the relative efficacy of the available PARPi in combination with durvalumab or nivolumab are currently under way in our laboratory.

The mechanism of action of PARPi includes: catalytic inhibition of PARP or “PARP trapping”, in which PARP is trapped at sites of DNA damage leading to prevention of repair and cytotoxicity. It has been postulated that PARP trapping activity may be indicative of higher efficacy in a PARPi [23]. The relative PARP-trapping activities of the PARPi used in this study vary as follows: talazoparib (100) > niraparib (2) > olaparib and rucaparib (1) > veliparib (<0.2), indicating that veliparib is almost purely a catalytic inhibitor, while talazoparib is primarily a PARP-trapper [41]. We found that PARP inhibitors with the highest PARP trapping activity such as niraparib and talazoparib were the most effective in enhancing the cytotoxicity of cisplatin against BLCA cells. These findings reveal that PARPi such as niraparib and talazoparib that have higher PARP-trapping activity may be efficacious as single agents against BLCA cells, while agents like olaparib and rucaparib that are mostly catalytic inhibitors may be effective in combinatorial regimen with cisplatin. Our results also indicated that combining PARP inhibition with cisplatin may allow a reduction in the effective concentrations of cisplatin needed for optimal anti-cancer effects and thus, potentially ameliorate some of the adverse effects associated with cisplatin-based therapy.

Defects in DDR can predict response to cisplatin-based chemotherapy in muscle-invasive BLCA [42]. Alterations in DDR genes such as ATM, RB1, and FANCC have been reported to serve as biomarkers of sensitivity to cisplatin-based chemotherapy. A recent study showed that PARP inhibition can lead to higher levels of DNA damage in combination with cisplatin compared with cisplatin alone [43]. The comprehensive genetic characterization of muscle-invasive BLCA has shown that ~34% of tissues harbor mutations in DDR genes such as ATR, MDC1, CHK1/2, ATM, BRCA1/2, and RAD52, suggesting that PARP inhibition can be effective in advanced BLCA [44, 45]. In line with these findings, our in-silico analyses revealed that ~30% of BLCA harbor mutations in HR genes. We found that the BLCA cell lines used in this study, UM-UC-3 and T-24 harbor mutations in HR genes such as ATM, ATR, RAD51, FANCD2, PRKDC, TP53, RECQL4, and WRN, which provides a rationale for using these cell lines to model responses of BLCA cells [26]. However, SV-HUC-1, used as a representative of normal urothelial cells, also harbors mutations in genes such as DNAH8 and BRCA1 [26]. Of the 3 cell lines used, SV-HUC-1 is the only cell line that harbors mutations in BRCA1, which may explain the suppressive effects of PARPi in these cells. While arguing for the suitability of cell lines to model tumor drug response, this also shows that caution is warranted in interpreting the observed responses. In summary, our study showed that combining PARP inhibition, especially the agents that trap PARP on DNA, can be efficacious in combination with cisplatin against advanced BLCA. While promising, these findings would need to be validated in large clinical cohorts.

## Conclusions

We demonstrated that PARP inhibitors can not only be effective against BLCA as single agents but can also be used in combinations to improve the efficacy of first-line therapeutic regimen. The current study is one of the first studies to compare all 5 commercially available PARP inhibitors and examine their relative outcomes in combination with cisplatin in BLCA. Given that PARPi are already undergoing clinical evaluation in urothelial bladder cancer [46], these pre-clinical results provide a rationale for testing the clinical efficacy of the PARPi+cisplatin combinations in BLCA patients.

## Supplementary Information

**Additional file 1.**

**Additional file 2.**

**Additional file 3.**

## Data Availability

All data generated or analyzed during this study are included in the published article and supplementary files.

## Abbreviations

BLCA: Bladder Cancer
DDR: DNA Damage Repair
PARP: Poly(ADP-Ribose)Polymerase
PARPi: PARP inhibitors
SSB: Single-strand Breaks
DSB: Double-strand Breaks
HR: Homologous Recombination
NHEJ: Non-Homologous End Joining
COSMIC: Catalogue of Somatic Mutations in Cancer
ATCC: American Type Culture Collection
TCGA: The Cancer Genome Atlas
NMIBC: Non-Muscle-Invasive Bladder Cancer
MIBC: Muscle-Invasive Bladder Cancer
FBS: Fetal Bovine Serum
SDS-PAGE: Sodium Dodecyl Sulphate-Poly Acrylamide Gel Electrophoresis
EMEM: Enhanced Minimum Essential Medium
SCID: Severe Combined Immunodeficient
