# Supplementary material for: PARP inhibitors chemopotentiate and synergize with cisplatin to inhibit bladder cancer cell survival and tumor growth
Source: BMC Cancer. 2022 Mar 23;22:312. doi: 10.1186/s12885-022-09376-9 (PMC8944004; doi:10.1186/s12885-022-09376-9)
Supplement: Supplementary file 1 — Additional file 1. [file 12885_2022_9376_MOESM1_ESM.docx]

Supplementary Figure 1: The BLCA cell lines UM-UC-3 and T-24 and the normal urothelial cells SV-HUC-1 were treated with sub-IC_50_ concentrations of niraparib, olaparib, rucaparib (5 μM each), talazoparib (0.5 μM) either singly or in combination with sub-IC_50_ concentration of cisplatin (0.5 μM) for 72 h. The resulting cell lysates were subjected to Western blotting with antibodies against the apoptotic markers cleaved/whole caspase 3, cleaved/whole caspase 9, or cleaved/whole PARP. Tubulin was used as the loading control. Representative full-length images of 3 independent experiments with duplicates are presented. The boxed areas represent the cropped images presented in Fig. 4. The table lists the catalog numbers, sources, clones, and dilutions of the different antibodies used for IHC and Western blotting.

Supplementary Figure 2: Sera from mice used in the in vivo tumor growth study were analyzed for the activities of alkaline phosphatase (ALP) and aspartate aminotransferase (AST) to assess hepatotoxicity. Results are presented as means ± SD of 5 mice in each group.
