# Supplementary material for: PARP inhibitors chemopotentiate and synergize with cisplatin to inhibit bladder cancer cell survival and tumor growth
Source: BMC Cancer. 2022 Mar 23;22:312. doi: 10.1186/s12885-022-09376-9 (PMC8944004; doi:10.1186/s12885-022-09376-9)
Supplement: Supplementary file 2 — Additional file 2. [file 12885_2022_9376_MOESM2_ESM.pdf]

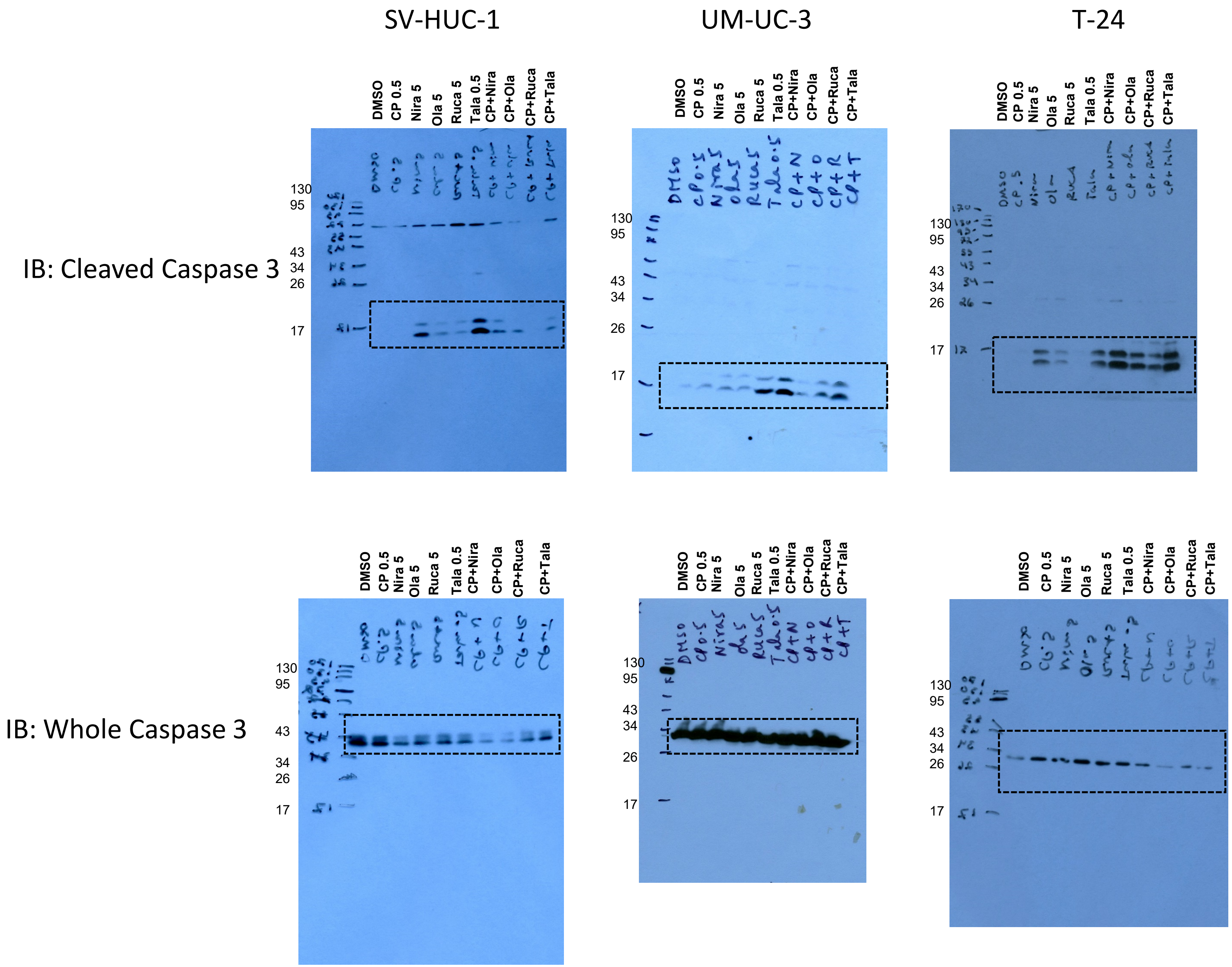
SV-HUC-1
UM-UC-3
T-24
IB: Cleaved Caspase 3
IB: Whole Caspase 3
DMSO
CP 0.5
Nira 5
Ola 5
Ruca 5
Tala 0.5
CP+Nira
CP+Ola
CP+Ruca
CP+Tala
130
95
43
34
26
17

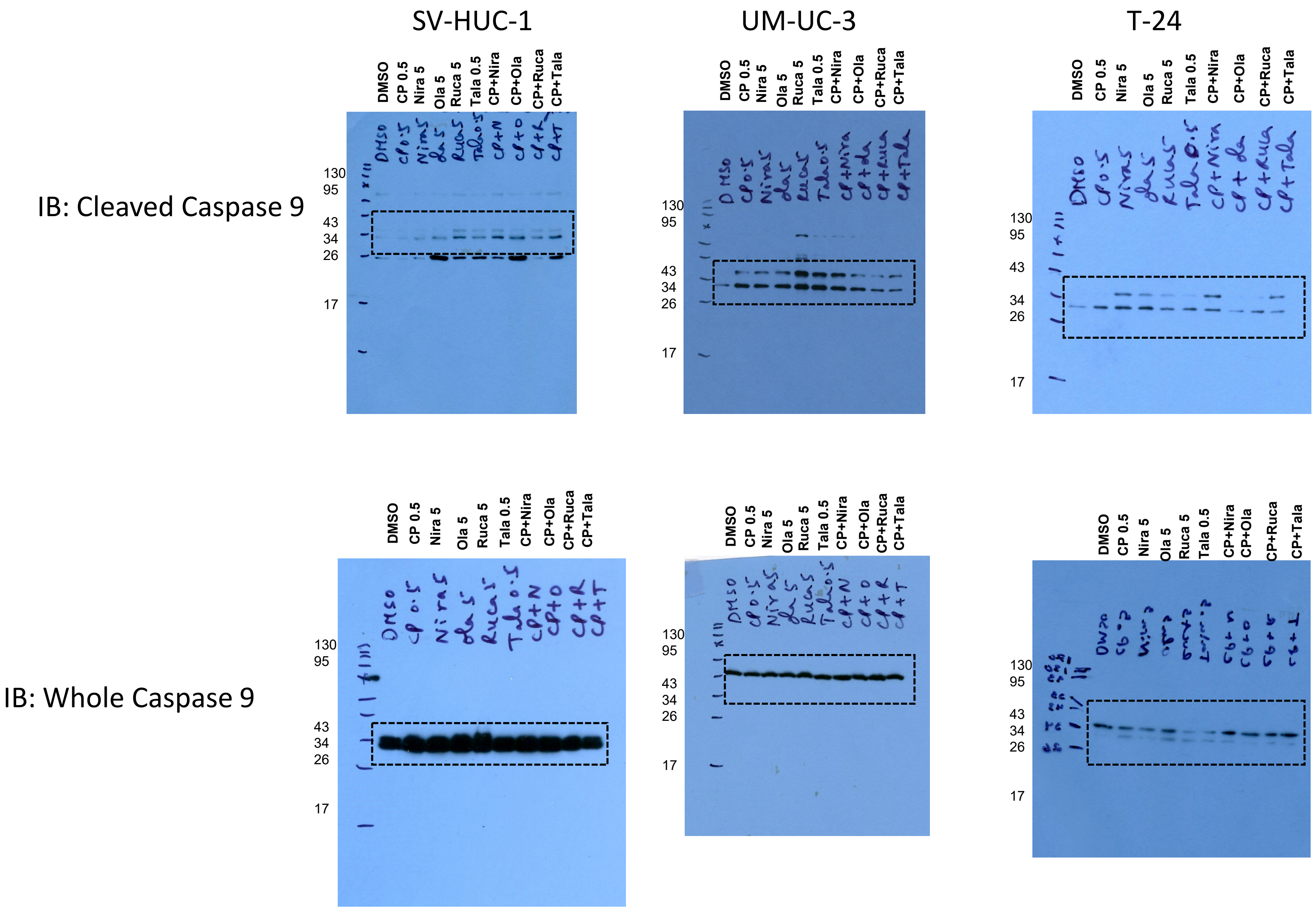
SV-HUC-1
UM-UC-3
T-24
IB: Cleaved Caspase 9
IB: Whole Caspase 9
DMSO
CP 0.5
Nira 5
Ola 5
Ruca 5
Tala 0.5
CP+Nira
CP+Ola
CP+Ruca
CP+Tala
130
95
43
34
26
17

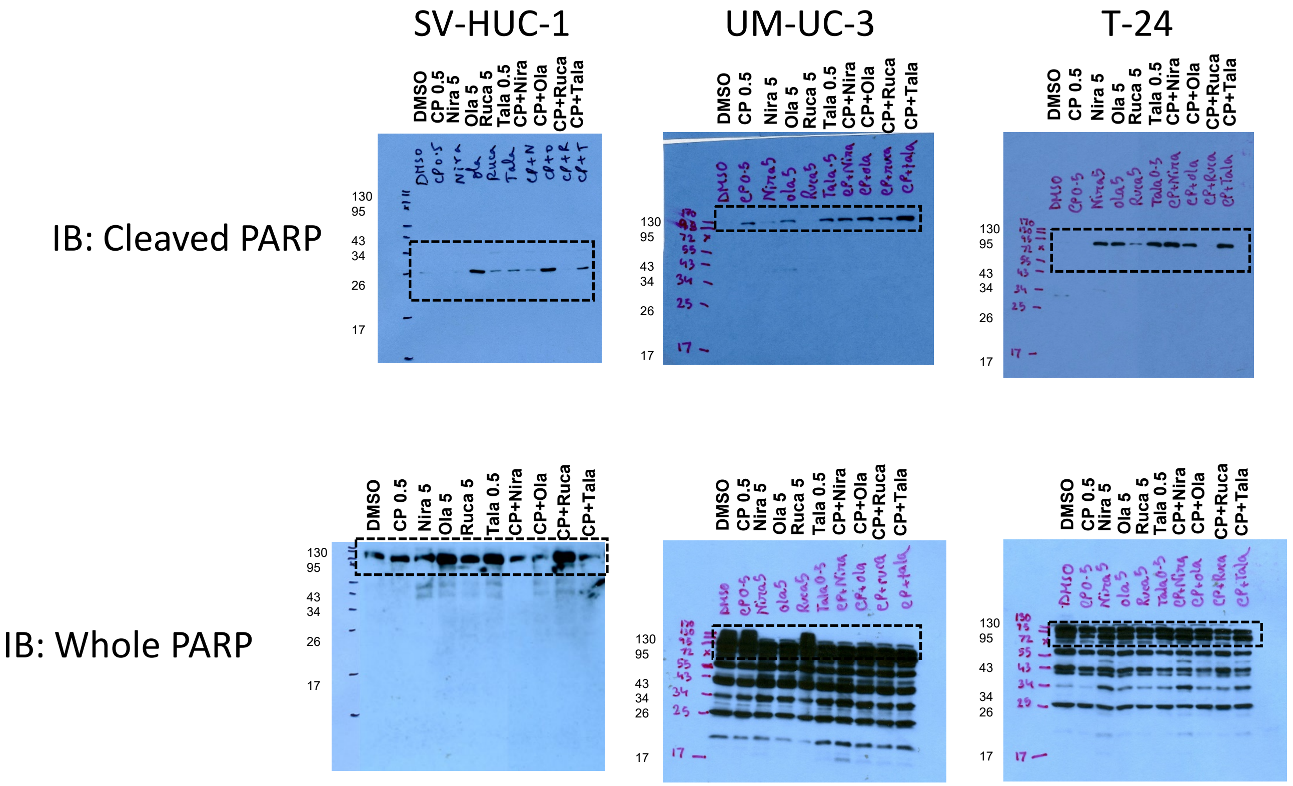

SV-HUC-1
UM-UC-3
T-24
IB: Cleaved PARP
IB: Whole PARP
DMSO
CP 0.5
Nira 5
Ola 5
Ruca 5
Tala 0.5
CP+Nira
CP+Ola
CP+Ruca
CP+Tala
130
95
43
34
26
17

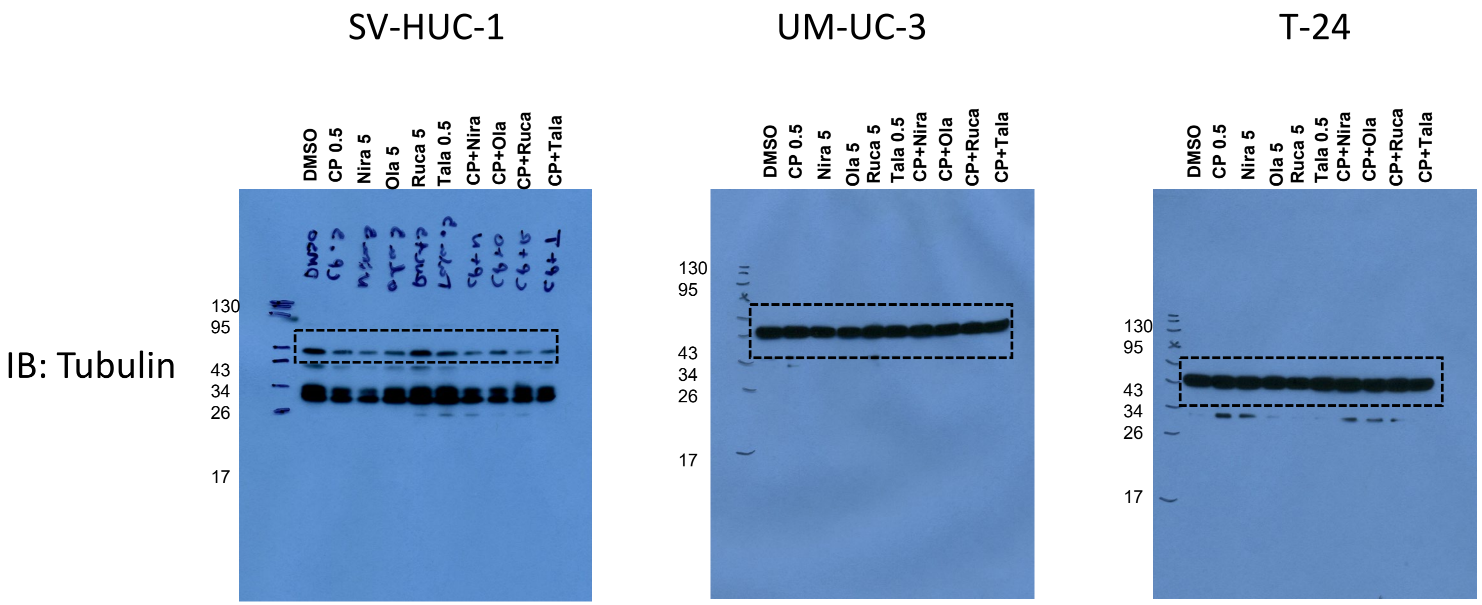
SV-HUC-1
UM-UC-3
T-24
IB: Tubulin
DMSO
CP 0.5
Nira 5
Ola 5
Ruca 5
Tala 0.5
CP+Nira
CP+Ola
CP+Ruca
CP+Tala
130
95
43
34
26
17

Antibody	Source	Cat#	Clone	Vendor	Dilution used for IHC	Dilution used for Western Blotting	Positive controls for IHC
Whole PARP	Rabbit	9542		Cell Signaling		1:2000	
Whole Caspase 3	Rabbit	14220	D3R6Y	Cell Signaling		1:1000	
Whole Caspase 7	Rabbit	12827	D2Q3L	Cell Signaling		1:1000	
Whole Caspase 9	Mouse	9508	C9	Cell Signaling		1:1000	
Cleaved PARP	Rabbit	5625	D64E10 XP	Cell Signaling		1:2000	
Cleaved Caspase 3	Rabbit	9664	D175 5A1E	Cell Signaling	1:500	1:1000	Prostate cancer cells treated with etoposide
Cleaved Caspase 7	Rabbit	8438	Asp198 D6H1	Cell Signaling	1:500	1:1000	Prostate cancer cells treated with etoposide
Cleaved Caspase 9	Rabbit	52873	Asp330 F5Z7N	Cell Signaling	1:500	1:1000	Prostate cancer cells treated with etoposide
Tubulin	Mouse	T8328	AA2	Sigma		1:10000	
Anti-mouse IgG, HRP-linked	Horse	7076P2		Cell Signaling		1:5000	
Anti-rabbit IgG, HRP-linked	Goat	7074P2		Cell Signaling		1:5000	
Ki-67	Rabbit	MA5-14520	SP6	Neomarkers	1:500		Breast cancer cell xenograft sections
