# Supplementary figures and images for: PARP inhibitors chemopotentiate and synergize with cisplatin to inhibit bladder cancer cell survival and tumor growth
Source: BMC Cancer. 2022 Mar 23;22:312. doi: 10.1186/s12885-022-09376-9 (PMC8944004; doi:10.1186/s12885-022-09376-9)

Suppl. Fig. 2

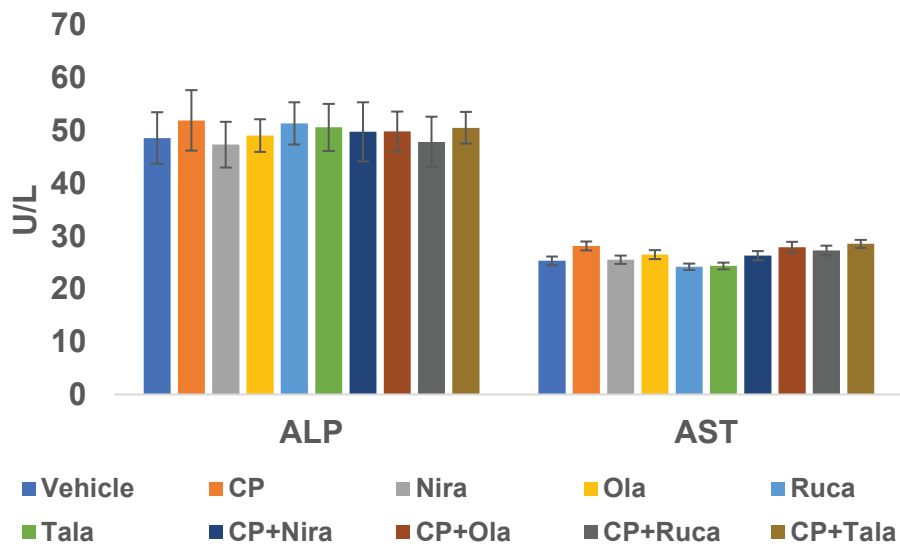

Supplement: Supplementary file 3 — Additional file 3. [file 12885_2022_9376_MOESM3_ESM.pdf]
